# Advances and New Therapies in Traumatic Spinal Cord Injury

**DOI:** 10.3390/jcm14072203

**Published:** 2025-03-24

**Authors:** Antonio Montoto-Marqués, Jesús Benito-Penalva, María Elena Ferreiro-Velasco, Mark Andrew Wright, Sebastian Salvador-De la Barrera, Hatice Kumru, Nelson Gaitán-Pérez, Agustin Hernández-Navarro, Antonio Rodríguez-Sotillo, Fernando Martins Braga, Angela Palencia-Vidal, Joan Vidal-Samsó

**Affiliations:** 1 Unidad de Lesionados Medulares, Complejo Hospitalario Universitario de A Coruña, Grupo de Investigación en Terapia Celular y Medicina Regenerativa, Instituto de Investigación Biomédica de A Coruña (INIBIC), 15006 A Coruña, Spain; 2 Fundación Institut Guttmann, Institut Universitari de Neurorehabilitació Adscrit a la UAB, 08916 Barcelona, Spain; jbenito@guttmann.com (J.B.-P.); mawright@guttmann.com (M.A.W.); hkumru@guttmann.com (H.K.); ahernandez@guttmann.com (A.H.-N.); fmartins@guttmann.com (F.M.B.); 3 Institut Universitari de Neurorehabilitació adscrit a la Universitat Autónoma de Barcelona, Bellaterra, 08193 Barcelona, Spain; 4 Fundació Institut d’Investigació en Ciències de la Salut Germans Trias i Pujol, 08916 Badalona, Spain; 5 Unidad de Lesionados Medulares, Instituto de Investigación Biomédica de A Coruña (INIBIC), Complejo Hospitalario Universitario de A Coruña, 15006 A Coruña, Spain; ma.elena.ferreiro.velasco@sergas.es (M.E.F.-V.); sebastian.salvador.de.la.barrera@sergas.es (S.S.-D.l.B.); nelson.gaitan.perez@sergas.es (N.G.-P.); antonio.rodriguez.sotillo@sergas.es (A.R.-S.); angela.palencia.vidal@sergas.es (A.P.-V.)

**Keywords:** traumatic spinal cord injury, neuroprotection, surgical treatment, cell therapies, spinal cord stimulation, robotic systems, exoskeletons

## Abstract

Recovery from traumatic spinal cord injury (tSCI) is challenging due to the limited regenerative capacity of the central nervous system to restore cells, myelin, and neural connections. At the clinical level, the fundamental pillars of treatment are the reduction in secondary damage (neuroprotection) and rehabilitation; these are the tools we have to mitigate the disability caused by spinal cord injury (SCI). To date, the treatments on which neuroprotection has been based are the prevention of acute respiratory failure to avoid hypoxia, early hemodynamic control, neuroprotective drugs and surgical management. Optimizing early hemodynamic control to ensure adequate spinal cord perfusion may be key to the management of SCI. While neuroprotective agents like methylprednisolone have fallen into disuse, several promising therapies are currently being tested in clinical trials. In terms of surgical treatment, although their impact on neurological recovery remains debated, appropriate early bone decompression followed by duroplasty in selected cases is increasingly recommended. Advances in cell therapies hold significant potential for enhancing both clinical and functional outcomes in SCI patients. Moreover, emerging neuromodulation techniques, such as transcutaneous and epidural stimulation, along with innovations in rehabilitation technologies—such as robotic systems and exoskeletons—are becoming indispensable tools for improving locomotion and overall mobility in individuals with SCI. This article provides an update on the advances in neuroprotection against secondary damage caused by tSCI, in cellular therapies, and in new rehabilitation therapies.

## 1. Introduction

Traumatic spinal cord injury represents a significant clinical challenge due to the lack of curative therapies and its limited potential for spontaneous recovery, making it one of the most devastating conditions for both patients and healthcare professionals.

The appropriate management of tSCI begins with the early diagnosis and timely transfer of the patient to specialized centers, emphasizing the importance of the “time is spine” concept [1], derived from the “therapeutic window”, which refers to the critical period after primary spinal cord injury during which it is possible to slow or limit secondary injury mechanisms, known as neuroprotection. While this approach highlights the importance of early surgery [2], it also emphasizes the need for the rapid transfer of tSCI patients to specialized centers, along with other essential therapeutic interventions such as prevention of acute respiratory failure secondary to SCI, which can induce hypoxia, and early hemodynamic monitoring, which is crucial to ensure adequate spinal perfusion. These elements are critical not only to improving clinical outcomes, but also to mitigating neurological damage and optimizing recovery.

In terms of pharmacological neuroprotection, several agents that modulate the immune system or inhibit key pathways involved in inflammation have been evaluated in the treatment of tSCI. These include non-steroidal anti-inflammatory drugs, cyclosporine, minocycline, erythropoietin and riluzole. Steroids, especially methylprednisolone, were previously the standard treatment, but are no longer generally recommended.

Experimental cell-based therapies, including mesenchymal stem cells and neural progenitor cells, are being explored for use in cell replacement and remyelination, along with strategies targeting neuronal repair, such as anti-Nogo A or monoclonal antibodies like elezanumab.

This review provides an update on the neuroprotective measures used in the management of tSCI. First, it will focus on early hemodynamic management aimed at optimizing spinal cord perfusion pressure (SCPP), along with current strategies for monitoring and improving SCPP to reduce secondary neurological damage. Additionally, it addresses the controversial role of surgical treatment, reviewing recent proposals on surgical procedures, timing, and their potential effectiveness, while highlighting the challenges in assessing their impact on functional recovery. On the other hand, although significant progress has been made with cell therapies, there is still a need to explore other emerging options, such as tissue engineering approaches and neurostimulation techniques. This paper reviews these therapies, most of which remain experimental but hold promise for advances in the treatment of tSCI.

Finally, there is evidence that rehabilitation, when applied in the right context, can play a critical role in regeneration and neuroplasticity. Therefore, new rehabilitation therapies such as robotic locomotor training, spinal cord stimulation and the use of exoskeletons that are changing the rehabilitation landscape in SCI are addressed. Current therapeutic strategies for traumatic spinal cord injury are summarized in Table 1.

## 2. Pathophysiology of Traumatic Spinal Cord Injury

Traumatic SCI occurs when the tissues surrounding and protecting the spinal cord (ligaments, muscles and bony structures) are unable to absorb the energy generated by the trauma. The spinal cord can be injured directly by the trauma or indirectly by the displacement of bone or disc fragments [3]. The most common mechanism of tSCI results from a combination of forces that cause violent movements of hyperextension, hyperflexion and axial compression of the head or trunk, resulting in bone and ligament injuries that eventually trigger SCI [4]. The magnitude and direction of the traumatic forces determine the type and extent of bone and ligament injuries, with the cervical spine being the most vulnerable due to its high mobility and low stability. In addition, there is a significant percentage of injuries that can occur without fracture or dislocation of the spine, such as injuries in children due to the greater flexibility of their spine, or in adults with significant degenerative changes. On the other hand, the complete anatomical transection of the spinal cord occurs in a small percentage of tSCIs [5].

Traumatic SCI is pathophysiologically divided into primary and secondary injuries [3]. As a result of the initial mechanical trauma, the spinal cord parenchyma is affected, with microhemorrhages disrupting blood flow in the central grey matter, leading to local infarcts due to hypoxia and ischemia. There is also a loss of nerve conduction in the adjacent white matter. This whole process is known as primary or immediate injury, and occurs within the first 0 to 2 h [6]. This is followed by the secondary phase. This is the consequence of a series of pathophysiological events triggered by the initial damage, consisting of an inflammatory cascade that destabilizes the axonal membrane, producing an irreversible pattern of spinal cord degeneration and neurolysis with necrosis of the neuronal and glial cells [3].

This secondary phase is divided into several stages: the acute phase (2–48 h), in which free radicals are released by lipid peroxidation of the cell membrane due to the initial trauma; spinal cord ischemia, associated with vasospasm with subsequent reperfusion, and the development of edema, which further aggravates the injury, and ionic disturbances. All this, together with a strong inflammatory response and cytokine release, leads to the apoptosis of neurons, oligodendrocytes, glial cells and astrocytes. During the subacute phase, which spans from 2 days to 2 weeks, increased phagocytic activity and astrocyte proliferation lead to the formation of a glial scar, thereby inhibiting axonal regeneration. Subsequently, in the intermediate (2 weeks to 6 months) and chronic phases, the glial scar matures and progressively extends the lesion, further obstructing axonal growth and contributing to the permanent loss of neuronal function [7].

## 3. Hemodynamic Management

The management of systemic hypotension and the optimization of mean arterial pressure (MAP) in the acute phase following tSCI have become critical strategies to enhance vascular perfusion and oxygen delivery to the spinal cord. However, given the complexity of local inflammatory responses and the intricate regulatory mechanisms governing spinal cord blood flow (SCBF) at the injury site, a treatment based solely on MAP may not be sufficient to ensure optimal spinal cord perfusion pressure. Consequently, emerging perspectives suggest that hemodynamic management should be more precisely tailored to optimize SCPP, rather than relying solely on MAP

### 3.1. Neurogenic Shock

Hypotension, defined as a systolic blood pressure (SBP) < 90 mmHg, is a frequent complication following acute SCI, and can arise from various etiologies, including hypovolemia due to hemorrhage, venous pooling in paralyzed muscles, bradycardia, and neurogenic shock. The cardiovascular dysfunction associated with SCI varies depending on the level of injury [8]. Injuries at the cervical and upper thoracic levels (above T6) are associated with more severe cardiovascular disturbances. 

Neurogenic shock represents a clinical manifestation of autonomic dysfunction, characterized by profound arterial hypotension and bradycardia, both of which increase the risk of secondary injury due to spinal cord hypoperfusion. It is crucial to distinguish neurogenic shock from hypovolemic shock, as the two conditions often coexist. Maintaining an SBP ≥ 90 mmHg is essential to mitigate the risk of further ischemic injury. The early and appropriate correction of neurogenic shock involves the administration of intravenous fluids to restore euvolemia, blood product transfusion when necessary, and, if required, the use of vasopressors to support cardiovascular function.

Given the potential for hemodynamic and autonomic instability, as well as the frequent occurrence of respiratory failure in the first week following SCI, continuous monitoring and intensive care management are essential [9].

### 3.2. Hemodynamic Management Targeting MAP

In the management of tSCI, controlling hypotension is crucial, but increasing MAP is also recommended. According to the 2008 Consortium for Spinal Cord Medicine, as well as the American Association of Neurological Surgeons (AANS) and the Congress of Neurological Surgeons (CNS) guidelines (2013), a target MAP should be maintained above 85 mmHg, and ideally within the 85–90 mmHg range, for the first seven days following SCI [10,11].

Although raising blood pressure has become a standard component of acute tSCI treatment, it is important to note that these recommendations are based on low-quality evidence. The definitive benefits of this approach in terms of neurological outcomes have not been conclusively established [12,13,14], and the implementation of these guidelines varies significantly across different regions worldwide [15,16,17,18,19].

A recent revision of the 2013 AANS/CNS guidelines led to the development of a new clinical practice guideline [9]. To inform this update, a systematic review of the literature was conducted, examining the relationship between MAP and changes in neurological function [14]. Based on the review and subsequent discussion, the committee chose to maintain the previous recommendations for the management of hypotension in acute SCI. Regarding MAP targets, it was proposed to maintain a minimum MAP of 75–80 mmHg and an upper limit of 90–95 mmHg during the first 3 to 7 days post-injury. Importantly, the upper MAP limit is recommended only for patients requiring active intervention to increase MAP. These recommendations are based on very low-quality evidence, and the strength of the recommendation remains weak, highlighting the uncertainty surrounding the neurological outcomes associated with specific MAP targets.

### 3.3. Effect of MAP Increase on Neurological Improvement

The relationship between MAP and neurological outcomes in SCI remains inconsistent in the literature. Several studies report that MAP values below 85 mmHg are associated with a lack of neurological improvement, while maintaining MAP ≥ 85 mmHg appears to correlate positively with neurological recovery [20,21,22,23]. This association is most evident in the first 2–5 days post-injury [20,22], although improvements may be observed in an even shorter time frame [20,23]. In terms of injury severity, the study of Catapano et al. [21] suggests that patients with ASIA Impairment Scale (AIS) grade A may benefit more from increased MAP that those with AIS D. However, other studies have found no clear association between MAP and neurological recovery [24], or have reported an optimal pressure range of 70–80 mmHg [17,20,25], which differs from the recommendations of the 2013 AANS/CNS guidelines.

Studies examining the impact of intraoperative MAP have shown that maintaining MAP within the range of 70–94 mmHg during surgery is associated with improved ASIA motor scores [26]. Additionally, using machine learning techniques, Torres-Espín et al. [27] identified an intraoperative MAP range of 76–104 mmHg as being associated with neurological recovery. Similarly, Agarwal et al. found that maintaining MAP between 80 and 96 mmHg was linked to better outcomes, while cumulative exposure to MAP outside the 76-(104–117) mmHg range for more than 93 min was associated with worse neurological function [28].

### 3.4. Variability of MAP in the Acute Phase of tSCI

An important consideration in the management of tSCI is the variability in achieving the pre-established MAP targets. Multiple studies have highlighted the challenges in maintaining target MAP levels, even in centers with expertise in managing acute SCI patients in neurocritical care units, and despite the use of vasopressors [16,20,22,29]. This difficulty has been attributed to the frequent occurrence of neurogenic shock and hypovolemic shock, particularly in polytrauma cases. Additionally, considerable variability in blood pressure has been documented during the critical phase of acute SCI [22,29], which can potentially influence prognosis [30].

In a prospective study by Kong et al. [31], which set a target MAP of 80 mmHg, 100% of patients had MAP readings below 80 mmHg at some point, and 81% had readings below 70 mmHg. Although measurements were taken hourly, the exact duration of these hypotensive episodes could not be determined.

Similarly, Gee et al. [29] analyzed minute-by-minute data from the first 5 days after SCI, and found that MAP fluctuated by approximately 3 mmHg per minute. In their study, MAP remained within the target range of 85–90 mmHg only 24% of the time, with the majority of recordings (57 ± 16%) exceeding 90 mmHg. These findings suggest that attempts to strictly adhere to target MAP levels may lead to overtreatment. Thus, while setting a target MAP is a common approach, it does not guarantee its achievement in all cases, nor does it ensure the prevention of episodes of hypotension or hypertension.

Collectively, these data suggest that hypertensive treatment should be prioritized early after acute tSCI. While studies do not establish a direct causal relationship between specific MAP targets and improved neurological outcomes, they indicate that neurological recovery may be associated with early MAP management, regardless of injury severity. Some studies propose that the duration of time spent below the target MAP threshold may have a more significant impact on neurological outcomes than the average MAP value itself. The primary goal is to control hypotension while avoiding sustained hypertension, due to the risk of hemorrhage and secondary tissue damage, particularly when vasopressors are required. Although a retrospective study of patients with cervical hemorrhagic contusion did not find a significant increase in the risk of hemorrhagic contusion expansion with elevated MAP levels [32], animal studies have demonstrated hemorrhage in the spinal cord when MAP is increased with vasopressor use [33,34]. Additionally, sustained hypertension may negatively impact neurological recovery [26,27]. The most recent guidelines for hemodynamic management propose maintaining a MAP range of 75 to 95 mmHg between 3 and 7 days post-injury. This approach allows clinicians to adopt a more individualized treatment strategy for acute tSCI patients, taking into account the severity of the injury, the patient’s overall health, and any comorbid conditions [9].

### 3.5. Pharmacological Agents for Increasing MAP

Maintaining MAP at supraphysiological levels carries inherent risks. Many patients with acute SCI require invasive monitoring and the administration of one or more vasopressors to achieve optimal blood pressure control. The use of vasopressors is particularly associated with cardiogenic complications, especially in elderly patients [35]. Despite their widespread use, there are currently no validated protocols to guide the selection of vasopressors in the hemodynamic management of SCI patients.

The 2008 Consortium for Spinal Cord Medicine guidelines recommend that vasopressor choice should be tailored according to the level of SCI [10]. Vasopressors that are α-receptor agonists increase blood pressure through peripheral vasoconstriction, whereas β-receptor agonists enhance cardiac contractility and heart rate. In patients with cervical and upper thoracic SCI, who often experience bradycardia and hypotension, a vasopressor with inotropic, chronotropic, and vasoconstrictive effects is typically required. In such cases, dopamine and norepinephrine—acting on both α1 and β1 receptors—are reasonable options. Pure α-agonists in these scenarios may exacerbate bradycardia [36].

In contrast, for injuries below T6, hypotension generally results from vasodilation or venous pooling, with sympathetic innervation to the heart often remaining intact. In these patients, a peripheral vasoconstrictor such as phenylephrine, which selectively targets α1 receptors to regulate peripheral vasodilation, is indicated. Dopamine or norepinephrine, which have both alpha and beta effects, in these cases may lead adverse effects such as tachycardia and atrial fibrillation [36].

Animal model studies suggest that norepinephrine may be more effective than other vasopressors in maintaining blood pressure and enhancing SCBF [33,34,37]. In an experimental study in pigs, norepinephrine was administered to gradually increase MAP, and its effects on SCBF were closely monitored. The results reveal that blood flow decreased when MAP was below 50 mmHg, whereas it increased significantly within the MAP range of 50 to 100 mmHg. However, increasing MAP above 100 mmHg did not lead to a further increase in blood flow. In a small prospective crossover study, Altaf et al. [38] evaluated the effects of norepinephrine and dopamine on intrathecal pressure (ITP) and SCPP in 11 patients with acute SCI. Both vasopressors achieved similar MAP targets; however, norepinephrine was more effective in maintaining MAP with lower ITP and higher SCPP compared to dopamine. These findings suggest that norepinephrine may be preferable to dopamine when vasopressor support is needed to sustain MAP in SCI patients. Other intravenous vasopressors for MAP support in SCI have limited clinical use [39,40]. Oral vasoactive agents like pseudoephedrine and midodrine may serve as adjuncts to intravenous vasopressors for increasing blood pressure and potentially reducing ICU stay [39,41]. However, further research is required before these agents can be recommended for routine use in acute SCI management [40,42].

Recent reviews on vasopressor support in SCI recommend the use of mixed adrenergic agents, such as dopamine and norepinephrine, for SCI above T6, while pure alpha-agonists like phenylephrine are suggested for lesions below T6 [39,40]. Given that dopamine is associated with a higher risk of cardiovascular complications, norepinephrine may be preferred for upper cervical and thoracic injuries [39], as it can increase SCPP while maintaining a similar MAP. The choice of vasopressor should be guided by the level and severity of the injury, the specific hemodynamic changes present, and a careful assessment of the risks and benefits, considering the patient’s age and comorbid conditions.

### 3.6. Hemodynamic Management Targeting on SCPP and Cerebrospinal Fluid Drainage

Hemodynamic management aimed at optimizing the SCPP has emerged as an innovative therapeutic strategy in the early stages of acute tSCI [43,44,45,46,47,48]. SCPP is defined as the difference between MAP and intraspinal pressure (ISP), often approximated by ITP [44], and is expressed as SCPP = MAP − ISP. This indicates that SCPP is not solely dependent on MAP. Consequently, a strategy that only adjusts MAP, without considering ISP, may not be sufficient to optimize SCBF in all patients.

Two methods for measuring ISP have been explored. The first involves the insertion of a pressure probe at the site of injury during posterior spinal surgery [43,45,47]. The pressure obtained through this technique is referred to as ISP, as it is believed to more accurately reflect the pressure within the spinal cord. The second method involves the percutaneous insertion of a catheter into the lumbar cistern, distal to the injury site, and is known as ITP or cerebrospinal fluid pressure (CSFP). This method measures the pressure exerted by the cerebrospinal fluid [45,46,48]. The advantage of the latter technique is that it is simpler to perform and allows for the potential drainage of cerebrospinal fluid if needed. However, its limitation is that it measures pressure below the level of the injury, and may not accurately reflect the pressure at the lesion site. Both methods carry potential complications, though several studies have demonstrated their safety [43,47,49].

Clinical research suggests that SCPP may serve as a more reliable indicator of neurological recovery than MAP. In a prospective observational study, Squair et al. [50] demonstrated that maintaining SCPP, measured as (MAP-CSFP), above 50 mmHg was a strong predictor of improved neurological outcomes. Subsequently, Squair and colleagues published findings from a study involving 93 individuals, which showed that maintaining an SCPP greater than 65 mmHg was linearly and positively correlated with the recovery of the total motor score at 12 months. The study also suggested that targeting this SCPP threshold was a more reasonable approach compared to using CSFP or MAP alone [51].

SCPP can be modulated through various interventions, including adjustments to MAP, CSF drainage (CSFD), decompressive surgery, or a combination of these approaches [46]. Assuming that ISP remains constant, an increase in MAP typically results in a corresponding rise in SCPP. However, the effects of vasopressors on ITP remain unclear. In a T5 SCI pig model, Martirosyan et al. [52] investigated the impact of increasing MAP, either alone or in combination with CSFD, on ITP reduction. In a subset of animals treated with phenylephrine via continuous infusion to elevate MAP, an increase in ITP was observed, along with a reduction in medullary blood flow. Similarly, Altaf et al. [38] reported an increase in ITP with dopamine administration.

In theory, ITP can be reduced by decreasing the volume of CSF in the spinal canal. Two strategies for CSFD have been described: reactive drainage, which is initiated only when SCPP falls below 65 mmHg, and empirical drainage, which involves draining 5–10 mL of CSF per hour regardless of the SCPP. Lavadi et al. [53] compared these two strategies and found that empirical CSFD, compared to reactive drainage, resulted in fewer cases of critical spinal cord hypoperfusion. However, the benefit of CSFD in tSCI remains unproven. Additionally, CSFD may be ineffective in cases of complete CSF obstruction. In the aforementioned study by Martirosyan et al. [52], raising MAP combined with CSFD resulted in a 24% increase in spinal cord blood flow.

In a randomized controlled trial, Theodore et al. [49] evaluated the safety and feasibility of CSFD used to improve SCPP in patients with cervical-level tSCI who had a lumbar drain placed following decompressive surgery. The control group received MAP elevation alone, while the experimental group received both MAP elevation and CSFD, targeting an ITP < 10 mmHg for 5 days. The CSFD group showed a reduction in mean ITP and a significant improvement in motor scores at 6 months compared to the control group. The authors concluded that CSFD is a safe and effective method for reducing ITP and improving SCPP in the acute phase following spinal cord injury. However, other studies have not demonstrated a significant reduction in ITP with CSFD using lumbar catheters [54,55].

Studies examining CSFD and SCPP in the acute phase of SCI have demonstrated their safety. However, data on their effectiveness in improving neurological outcomes remain limited. Some researchers suggest that targeting SCPP, rather than MAP, may be a more effective strategy for the acute management of SCI [43,56]. At Zuckerberg San Francisco General Hospital, the monitoring of SCPP through the placement of a lumbar subarachnoid drain for 5 days, with the goal of maintaining an SCPP ≥ 65 mmHg using intravenous fluids and vasopressors, has become standard practice in SCI care [56]. As further evidence regarding the effectiveness of this approach accumulates, more robust and reliable clinical recommendations can be formulated.

Maintaining SCPP is the primary goal in the hemodynamic management of acute tSCI. The most recent guidelines from AO Spine recommend avoiding an SBP drop below 90 mmHg, and suggest maintaining a MAP between 75 and 95 mmHg for 3–7 days post-injury (weak recommendation). However, a hemodynamic target aimed at SCPP (calculated by ISP or ITP) or a target aimed at ISP still requires validation through large-scale clinical trials. Emerging real-time monitoring techniques, such as microbubble contrast-enhanced transcutaneous ultrasound [57] and epidural near-infrared spectroscopy sensors for the spinal cord [58], are currently under development to better track changes in spinal cord perfusion. The heterogeneity of tSCI, in terms of clinical presentation, natural history, and demographic factors, suggests that a one-size-fits-all hemodynamic management strategy may not be feasible. The integration of artificial intelligence and machine learning, capable of processing large datasets, holds promise for enabling more personalized and data-driven approaches to treatment [59].

## 4. Surgical Approach

Given the significance of secondary injury in the pathophysiology of tSCI, the idea that surgical decompression could aid neurological recovery in a broad range of patients is particularly appealing to healthcare professionals, especially surgeons. However, the effectiveness of surgery for promoting neurological recovery in tSCI and the optimal timing for decompression have been controversial for many years, a debate that dates back to the early laminectomies performed by the Byzantine physician Paul of Aegina [60].

The general principles of spinal stabilization are well-established and have not changed significantly in recent years. There is broad consensus regarding surgical indications. While some injuries may be managed conservatively, surgical treatment is typically indicated to stabilize unstable vertebral injuries, reduce fracture-dislocations that cannot be corrected by closed reduction, and address late instabilities, usually due to failed conservative treatment or prior surgery. Emergency surgery is recommended in cases of neurological deterioration caused by compression from displaced bone or disc fragments, or epidural hematoma. Surgery is also indicated when conservative management fails to achieve spinal stability and neurological deterioration is likely.

### 4.1. Surgical Procedure

Traumatic SCI encompasses a wide range of severity, from mild to severe injuries, making a one-size-fits-all treatment approach unsuitable. The choice of surgical procedure must be individualized for each patient. The primary goals of surgery are to stabilize the spine and decompress the spinal cord. The decision on the most appropriate surgical procedure depends on factors such as injury severity, location, mechanism, extent of compression, and the surgeon’s preference. In cases of complete SCI, the overall prognosis is typically poor, and the primary surgical goal is spinal stabilization. For incomplete SCI, the surgical approach is determined by the injury mechanism and other relevant factors.

From the perspective of tSCI pathophysiology, prolonged spinal cord compression after the initial injury can exacerbate secondary damage by increasing edema and ischemia. This hypothesis is supported by several experimental studies, where histological analysis showed greater injury severity in cases with more significant canal narrowing and prolonged compression [61,62]. Therefore, it can be concluded that a surgical procedure that successfully decompresses the spinal cord could help minimize damage and facilitate neurological recovery after SCI. However, adequate surgical decompression is rarely verified via imaging. Furthermore, some authors, such as Aarabi B et al., postulate that the terms ’surgery’ and ’decompression’ have been used interchangeably in tSCI, with simple bony realignment and arthrodesis, with or without laminectomy, being accepted as resulting in spinal cord de-compression. Thus, in a retrospective study whose main objective was to evaluate complete cord decompression (defined as the presence of a permeable subarachnoid space around a injured spinal cord) by postoperative Magnetic Resonance Imaging (MRI), these authors found that conventional anterior cervical discectomy and fusion alone decompressed the injured spinal cord in only 46.8% of cases classified as grade AIS A and B. Similarly, corpectomy without laminectomy resulted in suboptimal decompression in 58.6% of these patients [63].

There seems to be agreement that effective decompression is the determining factor in the choice of surgical approach. In terms of what is considered adequate decompression, the presence of at least one spinal canal with CSF circulating circumferentially in the subarachnoid space around the spinal cord should be accepted as a minimum criterion. However, over the years, many surgeons performing this type of injury have adopted the belief that bony realignment and internal fixation, either with or without laminectomy, will produce a ‘decompression’ effect. However, this perspective often fails to take into account a detailed analysis of the extent of spinal cord inflammation, the nature of spinal cord compression, and the specific surgical techniques required to achieve an effect [64].

There are two main types of spinal cord compression: extradural and dural. Extradural compression occurs due to bony elements, either vertebral displacement or bone fragments, whereas dural compression is due to the dura mater restricting cord extension in situations of trauma-induced edema or hemorrhage. In a study by Saadoun et al. of 65 patients with tSCI and 15 controls without neurological involvement, 95% of patients were found to have spinal cord compression on MRI on admission. Of the patients with cord compression, 25.8% had dural compression and 74.2% had extradural compression. The authors concluded that the role of the dura mater in spinal cord compression is often underestimated. They suggested that bony realignment with laminectomy and instrumentation is unlikely to effectively decompress the affected spinal cord due to the persistence of dural compression. To effectively decompress the injured cord, they proposed that laminectomy combined with expansive duroplasty to provide additional space for the swollen spinal cord would be necessary; this approach is analogous to decompressive craniotomy with dural opening after traumatic brain injury [65]. In a prospective comparative study of 21 patients (11 with laminectomy and 10 with laminectomy and duroplasty), the same authors reported a more effective reduction in ISP and an increase in SCPP in those who underwent expansive duroplasty. This suggests that laminectomy alone may not be sufficient, and that expansive duroplasty may be necessary to reduce ischemia secondary to spinal cord compression [66].

In summary, some authors have recently advocated spinal decompression that includes durotomy and duroplasty in addition to bony decompression by laminectomy and arthrodesis. This strategy is even suggested for tSCI without radiological abnormalities (SCIWORA) [67]. We believe that this approach should be used in selected cases based on MRI findings. To investigate the role of duroplasty in patients with tSCI, a randomized, double-blind, multicenter, phase III clinical trial called DISCUS: Duroplasty for Injured Cervical Spinal Cord with Uncontrolled Swelling is currently underway (NCT04936620) [68].

### 4.2. Surgery Timing

The timing of decompression in acute tSCI has been controversial for many years, and remains so. If we recognize the importance of surgical treatment in tSCI to promote neurological recovery and prevent or mitigate secondary injury, known as neuroprotection, we would have to advocate early surgery. Several authors have investigated the time threshold that distinguishes between early and late surgery, but it is not yet clear how much time should be considered ‘early’. Some studies have suggested three time thresholds. According to Wilson [69], the times are as follows: ultra-early—8–12 h after injury; early—24 h; late—48–72 h. On the other hand, Aarabi [70] defines the following thresholds: ultra-early—<12 h; early—<24 h; late—>24 h.

Traumatic SCI is not limited to primary injury, but results in a process of secondary injury that begins immediately after the trauma [7]. This process involves a series of pathophysiological events triggered by the initial injury, as detailed in the pathophysiology section. However, the exact “therapeutic window” during which surgical intervention could most effectively mitigate secondary injury remains poorly defined. Although the 24 h threshold is commonly used in the literature to differentiate between early and late surgery [71], it is important to recognize that this time limit is largely arbitrary from a biological perspective; it has been set primarily for practical reasons, and could be even lower. As a result, there is considerable variability in surgical practice around the world. In fact, most current clinical guidelines are based on limited evidence and provide low-grade recommendations for performing surgical decompression in the first 24 h [72,73,74]. Recent AOSpine clinical guidelines recommend, with a moderate quality of evidence and a strong grade of recommendation, that early surgery should be offered as an option to patients with acute tSCI, regardless of lesion level. In this context, 24 h is considered to be the time threshold that distinguishes early from late surgery. However, the same guideline states that it is not possible to recommend ‘ultra-early’ surgery (less than 8–12 h) on the basis of the current evidence, due to the limited number of studies with small sample sizes, the lack of consensus on the definition of ‘ultra-early’ surgery, and the inconsistency of the available findings [71]. In support of this, the results of a prospective, observational, multicenter clinical trial (SCI-POEM) have recently been published, which aimed to assess whether early surgery (<12 h) leads to better neurological recovery, as measured by the lower limb motor score, compared with delayed surgical treatment (>12 h and <14 days). The trial included 291 patients, of whom 159 were assigned to the early treatment group and 135 to the delayed treatment group. The results show that early surgical decompression after acute tSCI did not result in clinically and statistically significant improvements compared to delayed surgical decompression at 12 months [75].

### 4.3. Neurological Recovery from tSCI After Surgical Treatment

The most debated and controversial aspect of surgical management in acute tSCI is the potential benefit in terms of neurological recovery. Early surgery aims to relieve spinal cord compression and reduce ischemia to improve conditions for recovery. Preclinical evidence suggests that spinal cord decompression reduces secondary nerve damage and improves outcomes [61,62]. A 2013 meta-analysis of 21 animal studies found that surgical decompression improved neurological outcomes by 35%. However, the authors caution about possible publication bias, as blinded assessments showed a significant reduction in the observed effect, which may have overestimated early decompression’s efficacy. Although these findings support early decompression as a promising strategy in animal models, translating them to human clinical practice remains complex due to uncertainties about its efficacy and optimal timing [76].

The AOSpine clinical guidelines, published in 2017, support the benefits of early surgery and suggest that early surgical intervention should be considered as an option for adult patients with tSCI, regardless of the level of injury. However, these recommendations are based on low-quality evidence and carry a weak strength of recommendation [72]. A very recent update of these guidelines recommends offering early surgery as a therapeutic option for adult patients with acute tSCI, regardless of the level of injury, with a moderate level of evidence and a strong grade of recommendation [77].

One of the most significant studies on the efficacy of surgical treatment for neurological recovery in tSCI is the STASCIS study (Surgical Timing in Acute Spinal Cord Injury Study) by Fehlings et al., published in 2012. This prospective cohort study involved 313 patients, of whom 182 underwent early surgery (<24 h) and 131 underwent late surgery (>24 h). The study concluded that surgical decompression within 24 h of injury can be performed safely, and is associated with a better neurological prognosis, as measured by at least a two-grade improvement on the AIS scale at the 6-month follow-up. However, the study has several limitations, including the exclusion of patients with severe concomitant injuries, a cohort limited to cervical spine injuries (where recovery potential is higher), and the fact that patients in the early surgery group were younger and had a higher percentage of severe injuries (AIS A and B), which may favor greater improvement. Additionally, 30% of patients were lost to follow-up, reducing the robustness of the results [78].

Recent systematic reviews and meta-analyses have provided important insights into the timing of surgery for tSCI. Qiu et al. analyzed 16 studies with 3977 patients comparing early surgery (<24 h) to delayed surgery, showing that early surgery led to greater neurological recovery. Specifically, patients who underwent early surgery had significant improvements of 2.3 points in motor scores, 5.1 points in light touch scores, and 4.4 points in pin-prick scores compared to those who had delayed surgery [79]. Another meta-analysis by Hsieh et al., which included 26 studies, found a significant association between early surgical decompression and at least a one-grade improvement on the AIS scale. However, the authors acknowledged the lack of high-quality evidence, and called for further analysis to confirm these findings [80]. A notable study by Badhiwala et al., analyzing data from four prospective, multicenter cohorts, also demonstrated improvements in motor, light touch, and pin-prick scores, as well as AIS grade, following early surgery. However, this study had several limitations, including the absence of randomization into early or late surgery groups, a 33% loss to follow-up, and potential inconsistencies due to variations in assessment methods and treatment protocols over the three decades included [81].

### 4.4. Evaluating the Effectiveness of Surgical Treatment in tSCI

A key issue in evaluating the effectiveness of surgical treatment for tSCI is the method used to assess outcomes, with most studies relying on the International Standards for Neurological Classification of Spinal Cord Injury (ISNCSCI) AIS grade and motor and sensory scores. Early neurological assessment is critical, as misclassification in the first 24 h could alter the AIS grade. Some guidelines recommend assessment at 72 h for more accurate results, as factors like spinal shock can affect initial examination accuracy. Burns et al. evaluated the reliability of the ISNCSCI examination within the first 48 h in AIS A patients, finding that factors such as mechanical ventilation, intoxication, head trauma, psychological disorders, and severe pain could affect reliability. Their results showed that patients with one or more of these factors were more likely to experience neurological improvement [82]. Evaniew et al. examined the impact of early neurological examination (within 48 h) on conversion rates in AIS A tetraplegia patients. They found that those assessed within the first four hours were significantly more likely to experience conversion from complete to incomplete injury, with conversion rates of 79% compared to 47% for those assessed later. Additionally, 50% of those assessed within four hours showed an improvement of at least two AIS grades, compared to only 21% of those assessed later [83].

An important consideration in evaluating motor and sensory scores on the ISNCSCI is the statistical significance of a four-point change in these scores. It remains uncertain whether this change reflects meaningful clinical improvement or is simply a statistical finding, potentially influenced by variability between researchers or even differences in assessments by the same examiner.

The effectiveness of surgery in neurological recovery following tSCI remains a topic of ongoing debate. While preclinical studies, the pathophysiology of secondary injury, and recent clinical research suggest that early decompression may improve neurological outcomes, its effectiveness cannot be definitively established due to significant heterogeneity across studies. These variations encompass differences in study design, methodology, surgical techniques, the timing of decompression, neurological assessments, patient cohorts, follow-up timing, and outcome measures, complicating the interpretation of findings.

In conclusion, although the value of early surgery in the management of tSCI is not in question, several critical aspects require clarification and standardization to enhance the strength of scientific evidence in this area. It is essential to define and standardize surgical protocols, determine what constitutes adequate decompression, and ensure rigorous postoperative evaluation. Furthermore, certain patient subgroups may derive greater benefit from surgery, while others may experience minimal improvement. Although the optimal timing for surgery remains uncertain, early intervention is generally considered reasonable, provided the patient’s stability allows it and that specialized surgical teams are available. Conversely, if surgery is delayed, the patient’s instability may significantly hinder timely intervention.

### 4.5. Intraoperative Ultrasound Imaging

Intraoperative ultrasound has become a useful tool in spinal cord tumor surgery, allowing the real-time visualization of the extent and precise location of the tumor. In addition, this technique facilitates the identification of residual lesions and involved vascular structures, which is essential to guide more effective resection. In this sense, ultrasound can not only help to reduce the risk of damage to surrounding nerve and vascular structures, but can also optimize the surgical process by improving the accuracy of tumor removal, which may benefit clinical outcomes [84].

Several studies have shown that intraoperative ultrasound is also useful in procedures such as cervical laminoplasty, allowing the real-time assessment of spinal decompression effectiveness [85]. Research using contrast-enhanced Doppler ultrasound in experimental rodent models of SCI has shown potential for assessing local hemodynamic changes in the injured spinal cord. This technique could be valuable in clinical practice, allowing the intraoperative evaluation of spinal vascular integrity and providing insights into how vascular changes affect functional outcomes after tSCI. This may improve surgical decision-making and post-operative management. Aarabi et al. also suggested that intraoperative ultrasound could complement laminectomy in the decompression of tSCI with expansive duroplasty, particularly in cases with severe inflammation and compression by the dura mater [86].

## 5. Pharmacological Neuroprotection

Pharmacological therapy in acute SCI has traditionally focused on addressing the cascade of pathophysiological events that contribute to secondary injury. This cascade, which begins within hours of trauma, encompasses inflammation, edema, ischemia, microglial activation, the release of free radicals and glutamate, lipid peroxidation, and demyelination [87,88]. For this purpose, various anti-inflammatory, neuroprotective, and axonal growth-promoting agents have been tested in both animal models and humans to mitigate the effects of secondary SCI. Some drugs, such as minocycline, nimodipine, tirilazad, and naloxone (with some studies involving combinations with methylprednisolone), have not progressed to further clinical trials. However, others—such as erythropoietin and progesterone combined with vitamin D—have shown promising results in reported studies [89]. Several compounds are currently under investigation in clinical trials, driven by their demonstrated efficacy in laboratory and animal studies of pathophysiology. Notable examples include riluzole, granulocyte colony-stimulating factor (G-CSF), and Rho protein antagonists, all of which are garnering significant attention [87,89,90]. However, the only group of drugs that have been widely used in clinical practice are the corticosteroids—in particular methylprednisolone (MP) sodium succinate [91]. The results of published clinical trials have been and continue to be the subject of debate and controversy [92].

Corticosteriods are known pharmacological agents with a neuroprotective function that acts by blocking Ca channels and preventing lipid peroxidation [90,93,94]. Previously used as an anti-inflammatory drug for brain tumors, a first trial of MP in 1984 established its safety and efficacy in acute SCI [95]. From the National Acute Spinal Cord Injury Study (NASCIS) II trial, conducted in 1990 in patients within the first 8 h of trauma, it was suggested that MP be infused over 24 h at a dose of 30 mg/kg in an initial bolus, followed by an infusion of 5.4 mg/kg/h over 23 h [96]. With the results of this trial and the subsequent NASCIS III trial—which prolonged perfusion by an additional 24 h if the patient was treated between 3 and 8 h after trauma [97]—the protocol for the use of high-dose MP in acute SCI became widespread in medical practice. However, subsequent analyses of published protocols and the results of other trials [98,99,100] showed that the resulting improvement in treated patients was not significant in the long term. In addition, a number of studies have reported the occurrence of adverse and potentially serious side effects in patients who received MP [98,101,102].

A detailed review by Hurlbert 2013 [103] showed the doubts of MP therapy and, finally, the clinical guidelines of the international expert societies did not include its recommendation in acute SCI [11,104].

However, subsequent reviews have suggested that, despite the limitations of the NASCIS II and III trials, some improvements can be inferred for patients receiving MP within the first 8 h. Although the initial Cochrane review was conducted by Bracken [105], a more recent analysis by Fehlings et al. [91] also examines the available literature and leaves open the possibility of continuing MP therapy in select cases. In his review of randomized trials, encompassing seven authors and protocols, Fehlings concludes that there is no evidence of neurological improvement in patients treated with MP according to the NASCIS protocol or similar regimens [91,106]. However, he does report a modest improvement in the motor index at 6 months in patients who received MP within the first 8 h, compared to untreated individuals. No significant increase in side effects was noted with the 24-h regimens, although a slightly higher incidence of gastrointestinal bleeding, wound infections, and pulmonary embolism was observed. In contrast, a substantial increase in the risk of pneumonia and sepsis was noted with the 48 h regimen. Based on this, Fehlings et al. [107] concluded that, while there is no evidence to suggest that MP treatment following the NASCIS II protocol leads to improved neurological recovery, moderate evidence points to a slight improvement in the motor index, with no significant differences in complications associated with the 24 h regimen.

Given these considerations, no formal recommendation has been established for the use of high-dose MP protocols—such as those in the NASCIS trials—for the treatment of acute SCI. The decision to administer MP within the first 8 h post-trauma, with a maximum 24 h infusion, remains at the discretion of the treating physician, provided informed consent is obtained from the patient.

Thus, over the past decade, the pharmacological treatment of SCI has faced a significant setback due to the absence of effective therapeutic agents. In recent years, alongside new revisions of MP treatment, there has been a renewed focus on the development of alternative compounds.

Progesterone administration has been tested in patients with traumatic brain injury. In this context, its neuroprotective effects are thought to be mediated through its action on receptors in the amygdala, hippocampus, and limbic system [89], as well as by inhibiting the expression of tumor necrosis factor alpha (TNF-alpha). It has also been used in experimental models of SCI [108], and in a clinical trial involving humans with acute SCI—where all patients received MP according to the NASCIS II protocol—the combination of progesterone and vitamin D appeared to have beneficial effects at 6 months [109]. Larger, more comprehensive studies are needed to further explore these findings.

Riluzole is another drug with neuroprotective properties, acting by reducing injury through the attenuation of glutamate excitotoxicity, blocking Na+ channels, and limiting neuronal death [110,111]. Following animal studies, a phase I trial conducted in 2010 demonstrated the drug’s safety and suggested some functional improvement, as a secondary endpoint, in treated patients [112]. A phase III trial was launched in 2013 but was prematurely halted due to the SARS-CoV-2 pandemic [113]. Although the results were not sufficiently powered for a definitive conclusion, they indicated improvements in both neurological and functional recovery in patients receiving the drug [110]. A recent systematic review with meta-analysis suggests that Riluzole is safe and leads to better neurological outcomes compared to controls in the treatment of tSCI, although these results did not reach statistical significance. The review concludes that more robust prospective and randomized studies are needed to further assess its safety and efficacy in tSCI [114].

G-CSF is a glycoprotein that stimulates the production of granulocytes in the bone marrow, and has recently been proposed to exert neuroprotective effects in animal models through multiple mechanisms [89]. Two studies—one phase I/II and the other phase II/III—investigated the effects of intravenous G-CSF infusion in patients with spinal cord injury [115,116], both yielding positive results. No significant adverse effects were observed. Larger, more well-powered studies are needed, potentially exploring G-CSF in combination with other therapeutic agents.

The potential to target axonal growth inhibitors has led to the exploration of Nogo-A protein blockers. These proteins play a key role in inhibiting axonal growth within the central nervous system, and blocking them with anti-Nogo antibodies may help counteract this inhibition, promoting axonal regeneration [89,117]. A recent review of animal studies identified 76 publications with varying results [118]. More recently, Maynard et al. conducted a phase I human trial involving a Nogo receptor blocker [119]. Other human trials are currently underway to further evaluate the therapeutic potential of this approach in SCI.

Another approach to neutralizing axonal growth inhibitors involves targeting Repulsive Guidance Molecule A (RGMa) [87]. This molecule binds to the neogenin receptor—a multifunctional transmembrane protein—and inhibits axonal growth by inducing apoptosis [120]. Preclinical studies in rodents and primates have been conducted using RGMa blockers, such as Elezanumab, a high-affinity, human-specific anti-RGMa monoclonal antibody. Elezanumab inhibits neogenin’s action and has demonstrated functional improvement in rodents following SCI [121], as well as in primate models [122]. A clinical trial is currently underway for patients with acute cervical spinal cord injury (EUCT number: 2023-505125-14-00).

In conclusion, pharmacological treatment for acute SCI remains under investigation, with ongoing randomized clinical trials aimed at demonstrating its efficacy. The absence of a universally effective drug should not be viewed as a failure. High-dose MP continues to be a therapeutic option in select cases, where, at the clinician’s discretion, it may offer benefits, provided that adverse effects are carefully managed. This includes patients with acute neurological deterioration or those presenting other factors suggesting a secondary neurological cascade injury. Given these considerations, further research into new compounds should be actively encouraged through multicenter trials, involving patient cohorts that are well-matched in all relevant aspects.

## 6. Stem Cells in Traumatic Spinal Cord Injury

Regenerative medicine is a transformative frontier in SCI research [123,124]. Recent breakthroughs in stem cell technology and delivery methods have turned these therapies from experimental concepts into plausible clinical solutions for SCI treatment, offering the potential to restore neurological function and improve the quality of life for millions worldwide [125,126].

### 6.1. Stem Cell Therapy: A Novel Frontier in SCI Treatment

Stem cells offer significant promise in treating tSCI due to their ability to self-renew and differentiate. These therapies aim to mitigate secondary injury, modulate the hostile post-injury microenvironment, and promote neuroregeneration, which could lead to functional recovery [125].

Stem cells exert therapeutic effects through two main mechanisms: (1) replacing damaged cells, and (2) modulating the injured microenvironment via paracrine activity. Through paracrine signaling, bioactive molecules like growth factors and cytokines help reduce inflammation, modulate the immune response, and support neuroprotection [125,127].

Both inflammation and glial scars play dual roles in SCI recovery. While early inflammation is necessary for debris clearance, prolonged inflammation worsens tissue. Similarly, glial scars protect surrounding tissue by isolating the lesion [128,129]; however, their dense structure and biochemical composition inhibit axonal regrowth and neural repair.

Preclinical studies have shown that stem cells can protect against secondary damage, repair myelin, and promote axonal regeneration, improving outcomes in animal models. However, translating these results to human trials remains challenging [130]. Clinical trials have yet to determine the optimal timing for stem cell transplantation, with most studies focusing on subacute or chronic patients due to practical considerations [131].

Clinical trials focus mostly on subacute or chronic SCI due to practical considerations, as the less mature glial scars in acute SCI and the potential for spontaneous recovery make it harder to evaluate stem cell therapy’s effectiveness. In chronic SCI, dense glial scars inhibit regeneration [128], but a stable functional baseline helps assess outcomes more accurately.

Challenges across all stages of SCI include the inflammatory and inhibitory microenviroment, immunogenicity, and the effective delivery and integration of stem cells. To address these barriers, strategies are being explored to achieve the following: (1) modify the post-injury environment, (2) target intracellular pathways to support neuronal regeneration, and (3) use stem cells to protect neurons, restore myelin, and enhance vascular repair. Achieving consistent success in human trials remains the next critical step [130].

Different stem cell types offer distinct potentials and limitations. The following section examines these cell types in detail, highlighting their roles in SCI treatment and ongoing research.

### 6.2. Types of Stem Cells

Stem cells are classified by their source, potency, and functionality. Embryonic stem cells (ESCs), derived from the inner cell mass of the blastocyst, are pluripotent [132], capable of differentiating into any cell type. Their high self-renewal capacity and minimal commitment make them promising for use in regenerative medicine [133], though ethical and immunological concerns limit their application [134]. Perinatal stem cells, collected non-invasively from umbilical cord blood and placental tissues during childbirth, are multipotent, and thus less committed, but still enable differentiation into various lineages. However, these cells do not raise ethical issues and, therefore, are increasingly utilized in personalized regenerative therapies [134].

Adult stem cells, including hematopoietic stem cells (HSCs) and mesenchymal stem cells (MSCs), have more restricted self-renewal and differentiation capacities compared to ESCs. However, their accessibility and adaptability make them widely used in autologous and allogeneic treatments [135]. Advances in stem cell technology have also introduced induced pluripotent stem cells (iPSCs), which are reprogrammed from somatic cells to mimic ESC pluripotency. Free from ethical concerns, iPSCs provide a versatile platform for disease modeling, drug testing, and personalized therapies [136,137]. Meanwhile, neural stem cells (NSCs), multipotent cells harvested from the brain or spinal cord, are being actively studied for their potential in treating neurodegenerative diseases and spinal cord injuries due to their ability to generate neurons and glial cells [138,139].

The next sub-sections examine the major stem cell types and their emerging applications in regenerative medicine, particularly for SCI. They also touch on combinational therapies that integrate diverse stem cell types with adjunctive strategies to address the challenges of clinical translation in SCI.

Table 2 summarizes key characteristics of the major stem cell types currently being explored for SCI treatment, highlighting their potency, therapeutic mechanisms, and clinical relevance.

#### 6.2.1. Mesenchymal Stem Cells

MSCs are the most widely studied stem cell types for treating tSCI [125]. They are versatile, with self-proliferation and multidirectional differentiation capabilities, making them valuable for regenerative medicine. MSCs can be isolated from almost all tissues, including bone marrow (BM-MSCs), human umbilical cord (hUC-MSCs), adipose tissue (AD-MSCs), Wharton’s jelly (WJ-MSCs), and amnion, and each source exhibits distinct cell surface markers [140,141,142,143].

These cells are highly viable, and provide structural support in SCI, making them seemingly the most therapeutic stem cells in this kind of lesion [144]. MSCs are easy to isolate and preserve, and raise minimal ethical concerns. They present immunomodulatory, tissue repair, neuroprotective, and angiogenic properties [141].

Due to their immune-privileged nature, MSCs do not require a close donor–recipient match. Furthermore, they release exosomes, numerous trophic factors and cytokines, improving the injured spinal cord’s microenvironment, inhibiting glial scarring and promoting cell regeneration [140,141,143].

The most commonly used methods of MSC transplantation include subarachnoid space transplantation, intravenous injection, and local intramedullary injection. Intravenous injection carries the risk of pulmonary embolism, while intrathecal injections require larger doses due to stem cell adsorption by the arachnoid membrane. Intramedullary injection allows direct delivery to the site of injury, but may increase tissue pressure and damage the surrounding spinal cord. Most clinical studies favor intrathecal or orthotopic delivery [140,141].

MSCs’ potential oncogenicity [140,141], the lack of an optimal transplantation strategy [145], and limited survival rates under hostile conditions remain important considerations. The timing of transplantation is critical. Transplantation during the acute phase risks creating a cytotoxic environment, whereas chronic-phase transplantation is limited by glial scarring, which inhibits axon regeneration [140].

Preclinical studies demonstrate MSCs’ efficacy [140], and early-phase clinical trials further support their safety and potential efficacy in chronic SCI individuals. A summary of these trials, including key parameters, is provided in Table 3 [146,147,148,149,150,151,152,153,154,155,156,157,158].

#### 6.2.2. Hematopoietic Stem Cells

HSCs can be harvested from adult bone marrow, placenta, and umbilical cord [159,160], with the latter being the preferred source due to its higher concentration and lower immunogenicity [160]. In the SCI microenvironment, HSCs promote repair by differentiating into macrophages, astrocytes, and neuroprotective glia, and by releasing cytokines that promote repair and suppress glial scar formation. Preclinical studies show functional neural recovery and motor neuron-like cell differentiation after HSC transplantation. Human studies to date have primarily focused on safety, with no significant adverse events reported. However, immune rejection remains a challenge for non-autologous HSC [159], and clinical efficacy data are limited [160].

#### 6.2.3. Neural Stem or Progenitor Cells

NSCs are multipotent cells that can differentiate into neurons, astrocytes, and oligodendrocytes, making them promising for SCI treatment [144,161]. They contribute to myelin regeneration, neural circuit reconstruction, and the secretion of trophic factors that support tissue repair and functional recovery [125]. Preclinical studies show improved motor and sensory outcomes after NSC transplantation, with early-phase clinical trials reporting encouraging results [161]. However, challenges like the hostile post-injury microenvironment, low survival and differentiation rates, and limited availability of clinical-grade NSCs persist [144]. Emerging strategies, such as biomaterial scaffolds and combinatorial therapies (e.g., pharmacological agents and rehabilitation protocols), are being developed for these issues [144,161].

#### 6.2.4. Embryonic Stem Cells

ESCs are pluripotent cells derived from blastocysts, capable of differentiating into neural and glial cells, making them promising for SCI treatment [125,162]. ESC-based strategies aim to replace lost neurons and promote tissue repair through the secretion of growth factors that facilitate tissue sparing, angiogenesis, and neural regeneration [162]. Preclinical studies show that ESC-derived oligodendrocyte progenitor cells can remyelinate axons and improve locomotor function in animal SCI [163]. However, their clinical translation is limited by ethical concerns and risks of immune rejection and teratoma formation [162].

#### 6.2.5. Induced Pluripotent Stem Cells

iPSCs are reprogrammed from adult somatic cells, providing an ethical and immunologically favorable alternative to ESCs [125,162]. iPSCs can differentiate into NPCs, which have been shown to integrate into host neural tissues, form functional synapses, and promote neuroregeneration, particularly in the acute and subacute phases of SCI [164]. However, challenges in clinical translation include genetic and epigenetic abnormalities, tumorigenicity, and immunogenicity [142].

### 6.3. Stem Cell Delivery Strategies

Effective stem cell delivery is critical to optimizing therapeutic outcomes in SCI, as it affects cell survival and integration at the injury site.

Less invasive delivery methods, such as intravenous administration, often result in poor grafting efficiency due to immune clearance and filtering by organs like the liver [165]. Localized delivery methods, such as intrathecal and intralesional (parenchymal) injections, offer a more targeted approach. Intrathecal delivery allows repeated, minimally invasive administrations [165]; however, the arachnoid membrane acts as a physical barrier, adsorbing transplanted cells and limiting their migration to the injury site, requiring higher doses to achieve therapeutic effects [140]. Intralesional injections deliver cells directly to the injury site, improving integration but carrying risks like increased tissue pressure and mechanical stress, potentially exacerbating secondary damage or hindering spinal cord perfusion [165].

To overcome these challenges, tissue engineering has introduced biomaterials, such as scaffolds and hydrogels, which mimic the extracellular matrix to create supportive microenvironments for stem cell therapies. These biomaterials enhance cell survival, engraftment, and therapeutic efficacy by facilitating bioactive factor release, providing structural stability, and promoting cellular interactions needed for regeneration [166].

### 6.4. Safety and Challenges

Key safety issues include tumorigenicity for pluripotent-derived cells like ESCs and iPSCs, and low survival rates for transplanted cells in the hostile post-injury microenvironment, where inflammation and glial scarring impede engraftment and neural repair [126,142]. Immune rejection also remains a concern for non-autologous stem cell sources, though certain types, such as WJ-MSCs, exhibit reduced immunogenicity [167].

Delivery methods also pose challenges. Intralesional injections, while allowing precise delivery to the injury site, risk causing additional tissue stress, whereas intrathecal injections require larger doses due to cell losses from arachnoid adsorption [126]. Moreover, ethical considerations surrounding clinical trial designs, such as the inclusion of sham-injected control groups, raise concerns about the risks associated with lumbar puncture [168].

A significant barrier to efficacy in chronic SCI is the formation of glial scars, which inhibit axonal regrowth and create a non-permissive microenvironment. Additionally, cavity formation at the injury site further complicates cell integration [164,169]. To improve outcomes, combinatorial strategies are being explored, such as combining stem cells with bioactive molecules or biomaterial scaffolds to promote cell survival and targeted therapeutic effects.

Finally, the emergence of stem cell tourism highlights ethical and regulatory challenges. Patients often pursue unverified stem cell treatments in unregulated jurisdictions, driven by anecdotal reports of miraculous recoveries that lack robust scientific evidence. These practices can undermine legitimate research efforts and pose serious health risks, including complications from poorly controlled procedures [170]. Addressing these safety and ethical concerns requires rigorous preclinical evaluation, transparent clinical trials, and increased education to guide patients toward validated treatments.

### 6.5. Ongoing and Future Trials

Building on advances in biomaterials and delivery strategies, ongoing research focuses on overcoming biological and technical barriers to enhance the clinical translation of stem cell therapies for SCI. A central approach involves improving the injury microenvironment by mitigating inflammation and reducing glial scarring to support regeneration.

Emerging cell-free therapies, particularly stem cell-derived exosomes, offer promising alternatives to direct cell transplantation. These extracellular vesicles carry bioactive cargo, including proteins, lipids, and microRNAs, such as miR-133b, that regulate inflammatory responses and promote tissue repair. Preclinical studies have demonstrated that MSC-derived exosomes can reduce inflammation, enhance axonal regeneration, and improve functional outcomes in animal models of SCI [145]. Importantly, exosomes mitigate the risks associated with tumorigenicity and immune rejection, making them safer and more stable for clinical applications [131].

Recent advancements include genetically engineered exosomes with tailored therapeutic payloads, designed to target specific pathways involved in apoptosis inhibition, cytokine modulation, and neural repair. These innovations provide greater precision and sustained therapeutic effects [131,145].

At the same time, tissue engineering innovations are refining biomaterial scaffolds and hydrogels to mimic the extracellular matrix, creating regenerative microenvironments that support stem cell therapies. These biomaterials provide structural stability, enhance cell survival, and enable the controlled release of bioactive factors essential for tissue repair and regeneration [131,164]. Hybrid approaches combining stem cells, biomaterials, and neurotrophic factors are yielding encouraging results in preclinical and early-phase clinical studies.

Future priorities include addressing scalability, regulatory approval, and the standardization of stem cell-derived products and delivery methods. Long-term follow-up studies will be essential to ensure the safety, durability, and reproducibility of therapeutic outcomes. With these advancements, stem cell therapies are increasingly positioned to transition from experimental treatments to transformative clinical solutions for SCI.

In conclusion, stem cell therapies represent a transformative frontier in the treatment of SCI, offering the potential to move beyond symptom management to actual neuroregeneration. Advances in stem cell research have expanded our understanding of their therapeutic mechanisms, including neuroprotection, paracrine effects, and axonal repair. Despite these promising developments, challenges such as tumorigenicity, immune rejection, and the non-permissive post-injury microenvironment persist, underscoring the need for combinatorial approaches that integrate biomaterials, scaffolds, and bioactive molecules.

Progress in delivery methods and innovations in cell-free therapies like exosomes highlight the evolving landscape of regenerative medicine. However, ethical concerns, scalability, and regulatory hurdles remain critical barriers to widespread clinical application. Ongoing and future trials, focused on optimizing safety, efficacy, and integration strategies, hold the key to unlocking the full therapeutic potential of stem cell therapies for SCI.

## 7. Spinal Cord Stimulation

### 7.1. Epidural Spinal Cord Stimulation: Motor Improvement Applications and Mechanisms in SCI

The motor function benefits of epidural spinal cord stimulation (eSCS) were first discovered serendipitously while treating intractable back pain in a multiple sclerosis patient [171]. Since then, eSCS has gained attention as a promising therapy for motor rehabilitation in SCI, enhancing recovery, voluntary movement, and functional independence [172,173,174,175,176,177].

Key clinical milestones highlight eSCS’s transformative potential. Harkema et al. [172] reported a motor-complete patient regaining full weight-bearing capability, while Angeli et al. [174] enabled motor-complete patients to perform overground walking. Gill et al. [175] documented independent stepping in an ASIA-A patient with task-specific training. Research shows that optimized stimulation can restore voluntary motor control in chronic SCI. Angeli et al. [173] activated lumbosacral networks to restore movement in four patients with complete paralysis. Wagner et al. [176] demonstrated that precise electrode placement accelerated motor recovery, with one patient regaining stepping ability in a week. Rowald et al. [177] reported rapid success using updated stimulation grids, enabling walking within a day.

eSCS enhances motor recovery by modulating spinal circuit excitability and engaging residual pathways. Stimulating the dorsal spinal cord activates proprioceptive afferents, influencing motor neurons and interneurons essential for movement [171,172,173,174,175]. This neuromodulation strengthens proprioceptive feedback and corticospinal connectivity, helping SCI patients regain voluntary motor control [171,172,173,174,175,176]

In conclusion, continued advancements in stimulation protocols and device technology, coupled with task-specific rehabilitation strategies, hold great potential to further improve patient outcomes and redefine the prospects for functional recovery in motor-impaired populations.

### 7.2. Transcutaneous Spinal Cord Stimulation in Individuals with SCI

Transcutaneous spinal cord stimulation (tSCS) is a non-invasive neuromodulation technique that stimulates spinal circuits via skin-placed electrodes [178,179,180,181,182]. It serves both as a tool to study spinal interneuronal function and as a potential rehabilitation aid for neurological disorders like SCI [180,183,184,185]. tSCS activates spinal networks by recruiting large-to-medium afferent fibers in the posterior root, increasing excitability without directly triggering action potentials [186,187]. This modulation adjusts spinal interneuronal excitability [179,180,181,182].

Additionally, cutaneous mechanoreceptor activation near electrodes may enhance neuromodulatory effects via polysynaptic connections [188]. In animal models, [189] found that both dorsal epidural SCS and tSCS over C3-C4 and C7-T1 potentiated supraspinal-evoked responses, with stronger effects in epidural SCS.

tSCS promotes recovery by neuromodulating spinal networks above, within, and below the lesion, enhancing voluntary motor control. Repeated sessions may induce adaptive processes, driving neural reorganization [190]. These activity-dependent changes can persist for minutes to days, supporting respiratory network reorganization and functional recovery post-SCI [190].

#### 7.2.1. tSCS Effect on Upper Extremity

Approximately 50% of individuals affected by SCI have lesions in the cervical region [191]. The loss of arm and hand function profoundly impacts their independence and quality of life.

Since epidural electrical stimulation proved effective for motor recovery [192], most research has focused on lower limb and postural control. Recent advancements have extended this to upper limb function. Lu et al. [193] reported improved hand strength and voluntary control in two chronic complete SCI patients after sSCS at C7-C8, with increased electromyographic activity and better clinical scores. Non-invasive cervical tSCS also showed promise, improving voluntary hand control in eight chronic SCI patients after one session, with grip strength increasing by 300% with stimulation and 200% without after four weeks [193]. Two-level stimulation (C3-C4, C6-C7) outperformed single-level stimulation.

Case studies highlight tSCS’s potential. In one report, 14 sessions of 0.2 Hz tSCS improved corticospinal excitability and motor-evoked potentials, though voluntary hand control gains were modest [194]. Another AIS D patient doubled their grip strength and dexterity over four weeks of tSCS with physical therapy, with sustained benefits at three months. Freyvert et al. [195] combined tSCS with buspirone, reporting significant gains, though buspirone’s specific effects remain unclear.

Crossover studies by García-Alén et al. [183] and Inanici et al. [185] explored tSCS combined with task training and robotic exoskeletons in cervical SCI patients. Using dual cathode electrodes at or below the injury level and a biphasic or monophasic waveform at 30 Hz, tSCS significantly improved upper limb strength, prehension ability, and pinch force compared to task training alone.

In a recent large-scale study, Moritz et al. [196] evaluated the safety and efficacy of tSCS in 60 chronic cervical SCI participants over 24 sessions. Stimulation at 30 Hz with a 10 kHz carrier frequency improved strength, fingertip pinch force, hand function, and quality of life for 72% of participants, with no serious adverse events reported. These findings suggest that tSCS, particularly when paired with rehabilitation, offers significant potential for enhancing upper limb function in SCI patients.

#### 7.2.2. tSCS Effect on Respiratory Function

Impaired respiratory function is a common consequence of cervical SCI, and may also occur after thoracic injuries [197]. Respiratory complications affect 84% of cervical [C1-C4], 60% of C5-C8, and 65% of thoracic [Th1-Th12] injuries.

Two key studies highlight tSCS’s impact on respiratory function. Gad et al. [198] treated a 39-year-old man with complete C5 SCI using cervical tSCS (20 min, twice daily) combined with respiratory muscle training for eight weeks. Biphasic pulses (10 kHz carrier at 30 Hz) were applied at C3-C4, C5-C6, or Th1-Th2, leading to significant improvements in inspiratory/expiratory pressure, forced vital capacity, dyspnea, and cough effectiveness.

Kumru et al. [199] studied 22 cervical SCI patients, comparing tSCS + inspiratory muscle training (IMT) versus IMT alone. The tSCS + IMT group (30 min daily, five days/week at C3-C4 and Th9-Th10) showed significant improvements in respiratory function, thoracic muscle strength, and hypophonia, unlike the IMT-only group.

#### 7.2.3. tSCS Effect on Trunk Function

Postural rehabilitation is a key priority for optimizing recovery in individuals with SCI. While tSCS shows promise for improving trunk stability, it remains in early development [200,201].

Five studies have assessed tSCS’s effects on trunk control in adults [186,200,201,202] and one in children [203]. Rath et al. [200] examined eight chronic SCI patients (C3-T9, AIS A or C) using monophasic rectangular pulses (1 ms, 30 Hz at T11 and 15 Hz at L1) at motor subthreshold intensity. A single session of exercises (1–2 min each, with 2 min rest intervals) enhanced external oblique and thoracic erector spinae activity, reduced pelvic retroversion, improved spinal alignment, and increased upper limb stability, range of motion, and movement speed during leaning tasks.

Sayenko et al. [186] studied 15 chronic SCI patients (AIS A–C) unable to stand independently. Six participants underwent 12 tSCS sessions (1 ms pulses at 15 Hz applied to L1–L2, 120 min/session, 3 times a week) targeting postural adjustments. Significant improvements in standing balance were observed with and without stimulation.

tSCS at T11-L2 has been linked to improved static and dynamic balance [201], trunk extension, upright sitting posture, transfer ability [203], and unilateral reaching [200]. However, Kumru et al. [202] found no significant changes in trunk stability when tSCS at C3-C4 or C6-C7 was applied during upper limb therapy in 13 cervical SCI patients over eight days.

These findings suggest that integrating tSCS with postural rehabilitation may enhance trunk control in SCI patients, though further research is needed.

#### 7.2.4. tSCS for Motor and Gait Recovery and Spasticity Management

tSCS delivers high-intensity electrical impulses (up to 200–250 mA) via transcutaneous electrodes over the vertebrae. Common cathode sites include C5, T11–L1, and Coc1, with T12-L1 frequently used for its link to central pattern generators [204]. Multisite stimulation enhances muscle activity and motor performance more than single-site [178,199]. Cervical tSCS increases cortical excitability, modulates spinal reflexes, and improves motor function, potentially reactivating dormant descending pathways [178,205,206,207]. Lumbar stimulation (L1) concentrates high current densities along the cauda equina and can induce step-like movements, leveraging the rhythmogenic properties of the lumbosacral cord [178,208,209].

Kumru et al. [199] reported that multisegmental tSCS improved walking time and muscle strength in SCI patients, with superior effects when applied at cervical, lumbar, and coccyx levels compared to single-site stimulation. Early studies on tSCS and gait recovery showed improved hip flexion, knee coordination, and reduced manual assistance in AIS D patients [204,210]. Gerasimenko et al. [178] found that 30 Hz tSCS induced stepping movements in healthy individuals and rhythmic leg movements in SCI subjects, with greater effects when paired with exoskeleton training.

Larger studies have reinforced tSCS’s benefits for gait. McHugh et al. [211] reported significant gains in walking speed and endurance, while Shapkova et al. [212] found improved foot loading, gait symmetry, and ambulation with combined tSCS and exoskeleton training. Estes et al. [213] showed enhanced walking outcomes in a stimulation (50 Hz) group, and Meyer et al. [214] found improved ankle dorsiflexion and walking speed. Kumru et al. [199] observed increased tibialis anterior MVC and reduced spinal excitability after multisegmental tSCS.

## 8. New Technologies Applied to Rehabilitation of Spinal Cord Injury

### 8.1. Robotic Systems and Exoskeletons for Gait Rehabilitation

A key goal of SCI rehabilitation is restoring walking ability, and various robotic systems are now available to support gait training. In line with the definitions drawn by Stampacchia et al. [215] and Midik [216], the robotic systems and devices studied for use with individuals with SCI can be separated into: (1) Robot-Assisted Gait Training systems (RAGTs); and (2) overground robotic exoskeletons (OREs) [215].

#### 8.1.1. Robot-Assisted Gait Training Systems

RAGTs are static robotics systems that were developed to include a body-weight support (BWST) system with an external robotic gait orthosis used in combination with a treadmill [216]. The distinct advantage of RAGT systems is that they allow a higher intensity of gait training, whilst still preserving a physiological gait pattern with significantly reduced physical demands and costs for the therapist [217]. The most widely used and recognized RAGT system is the Lokomat^®^ (Hocoma; Zurich, Switzerland) [217]. Extensive research has been conducted in the SCI population using the Lokomat^®^, with notable benefits in persons with incomplete SCI reported that include improvements in gait distance, restoring of gait function, the balance and symmetry of the walking pattern, and improved outcomes in gait measures such as the Walking Index Spinal Cord Injury (WISCI) compared to conventional rehabilitation [215,216,218,219]. Furthermore, due to the highly supportive design of the system, the Lokomat^®^ has been used in studies with persons with SCI as high as C1; however, the secondary benefits of reducing pain and spasticity and improving cardiovascular function are still unclear [215,220]. A significant drawback of these RAGT systems is that they are not movable, with gait training being restricted to the BWST. However, RAGT systems hold a significant position in clinical rehabilitation centers, as they allow a wide variety of patients to receive gait training with low risks of adverse events when compared to other robotic devices for gait rehabilitation [221]. It has been suggested that an avenue of future use will rely on hybrid designs of RAGT systems that integrate user-specific feedback, brain–computer interface technologies and adaptive support, creating a more holistic treatment for the user [222].

#### 8.1.2. Overground Robotic Exoskeletons

As the name would suggest, OREs are devices that can be used for overground training in a variety of environments, and are not restricted to being solely in one location [215]. Within the umbrella of OREs, there is a spectrum of differences between the weights, sizes, orthotic designs, and methods of activation [223].

Of the OREs that are most widely known and used within clinical settings for SCI rehabilitation, there are generally two types of devices—crutched and crutch-less OREs [224]. Crutched OREs such as the ReWalk™ (ReWalk Robotics Inc., Marlboro, MA, USA), Ekso^®^ (Ekso Bionics, Berkley, CA, USA) and the Indego™ (Parker Hannifin Corporation, Cleveland, OH, USA; Part of Ekso Bionics, San Rafael, CA, USA since 2022) are devices that provide support from the hips to the feet, with motorized joints at the hips and knees that work in the sagittal plane and require the user to use a walking aid [225]. These devices are adjustable to a user’s size within an anthropometric limit, whilst Indego™ has the added feature of being created using a modular design for more accessible donning and doffing [226]. These OREs are lightweight compared to other types of exoskeletons, weighing between 12 kg (Indego™) and 27 kg (Ekso^®^) [226,227]. The majority of these crutched OREs utilize an activation method to trigger steps that rely on sensors that detect the user’s movement in the trunk, pelvis or hips to activate a step [225]. The crutched OREs ReWalk™ and Indego™ have been approved for use in clinical settings for those with a T4-L5 AIS A-D injury, and in community settings for those with a T7-L5 AIS A-D injury. The Ekso^®^ has been approved for clinical use under trained supervision for those with a T4-L5 AIS A-D injury, and as high as C7-L3 in those with an AIS D injury [228].

The HAL^®^ (Hybrid Assistive Limb) (Cyberdyne, Tsukuba, Japan) is a unique crutched ORE that incorporates electromyographic sensors to monitor bioelectrical signals from innervated muscles of the user. These sensors detect bioelectrical signals, which then trigger the exoskeleton to move based on the user’s intent [229]. Recent studies have shown that the use of the HAL^®^ in SCI rehabilitation can support gait recovery in individuals with SCI [230,231]; however, studies with high-level evidence are still lacking, particularly given the reliance on detectable electromyographic signals to activate the system, which limits the accessibility for those with motor complete lesions [229]. Previous studies have shown success when using upper limb activity as the trigger for the device in those with complete injuries; however, this has been tested in a small number of users, and is not yet generalizable [230,232]. Besides these devices, there are crutch-less OREs that do not require the user to have a walking aid, and include devices such as the Atalante-X^®^ (Wandercraft, Paris, France) and the REX^®^ (RexBionics, Auckland, New Zealand) [224]. These devices are heavier (Atalante-X^®^, 80 kg; REX^®^, 38 kg) OREs that extend from the trunk to the feet, and can be adjusted to the user’s size and require no support via walking aids to balance [224,233]. Notably, they can contain between 10 (REX^®^) and 12 (Atalante-X^®^) motors across the hips, knees, and ankles that allow the user to move forwards and backwards in the sagittal plane and sideways in the frontal plane, as well as performing other rehabilitation activities such as turning and squats [224,233]. The REX^®^ is operated by the user via a joystick control that requires minimal upper limb functionality, whilst the Atalante-X^®^ has pre-programmed walking and standing modes that the user triggers by moving the trunk forwards and activating the Inertial Measurement Unit sensor [224,233]. Following a study by Kerdroan, as of 2024, the Atalante-X^®^ has been approved for use in rehabilitation for persons with complete SCI at T5-L5 [224]. In the RAPPER II trial, the REX^®^ was shown to be safe and effective for SCI rehabilitation in 56 persons with complete or incomplete SCI from C4 or below, and has been registered for use under the supervision of a healthcare professional in clinical settings [233]. In contrast to other exoskeletons, this study showed that it was possible to train upper-body exercise, trunk control, balance and gait in the REX^®^ device [233].

Despite advancements, OREs face challenges in terms of clinical adoption, cost, and limited evidence, which restrict its full potential. Current research highlights benefits for walking support and secondary health conditions in individuals with SCI. However, gaps in understanding body interaction, dosage effects, and adverse outcomes persist. With continued innovation and rigorous studies, including more RCTs, OREs hold promise for enhancing rehabilitation and expanding accessibility for SCI patients.

### 8.2. Virtual Reality Systems in SCI Rehabilitation

Virtual reality (VR) is a technology that allows users to be immersed in three-dimensional virtual worlds using devices such as head-mounted displays (HMDs), tracking systems, sound equipment, and haptic-feedback gloves [234]. Systems for VR can vary from non-immersive setups that involve basic monitors for the user to fully immersive experiences through HMDs that completely engage users [235]. Moreover, VR has shown potential in managing neuropathic pain by immersing patients in environments that distract them from pain sensations [236]. It is hypothesized that the use of VR can promote neuroplastic changes through increased task repetition, with the gamification available via VR making tasks competitive, and with real-time feedback resulting in improved output and alleviating the potential for boredom and disengagement following repetitive task practice [234,237].

A systematic review of 16 studies published in 2024 highlighted that there were significant differences in favor of VR-based rehabilitation for outcomes on the WISCI and BERG Balance Scale in comparison to conventional therapy alone. However, the findings for the 10 MWT, TUG, Box and Blocks and LEMS were all non-significant [234]. Previous studies have shown a high dropout rate for VR, whilst others point towards a lower acceptance rate compared to conventional therapy, which may be due to the provocation of adverse symptoms such as vertigo and headaches, and difficulties with orientating oneself with the VR headset [234,235]. Additionally, the cost and complexity of the VR can also be a limitation to the accessibility of use, with therapists also needing to receive training to be able to setup, adapt and support the user during the rehabilitation session [235].

Although current evidence on the long-term efficacy of VR in SCI rehabilitation is limited by small sample sizes and a lack of RCTs, existing findings highlight its potential to enhance physical, psychological, and functional outcomes as a supplement to conventional therapy. Improving VR systems to more closely match the motor and cognitive capabilities of individuals with spinal cord injuries by increasing immersion, multisensory interaction, and user engagement—while maintaining affordability—could enhance both accessibility and effectiveness [234,235,236].

### 8.3. Wearable Sensors and Monitoring Technologies

The use of wearable sensors and monitoring technologies in SCI rehabilitation has developed over the past few years, with studies conducted to monitor the physical activity of patients [238]. These devices, including accelerometers and gyroscopes, offer precise feedback on physical activities such as gait patterns, posture, and upper limb movements, allowing clinicians to create personalized, adaptive treatment plans [239].

The benefits of wearable sensors in SCI rehabilitation are significant. They provide objective, long-term data that support better diagnostic decisions, improve monitoring in habitual environments, and enhance the independence of individuals with mobility impairments [239]. Their ability to deliver tailored feedback also improves outcomes in areas such as strength training, gait retraining, and activity detection [240]. An additional benefit of these technologies is that they empower patients by enabling remote rehabilitation, and can reduce the need for frequent clinical visits [241]. However, these technologies are not without challenges. Risks include data privacy concerns, potential inaccuracies in data collection, and discomfort or irritation from wearing the devices [240,242]. Technical limitations, such as the need for calibration, signal interference, and the risk of data overload, may further complicate their use [243].

Currently, there is a lack of studies specifically on the use of these technologies within SCI rehabilitation; however, there is hope that they can provide a versatile and efficient means of tracking recovery progress, enabling clinicians to address patient needs more effectively. As the technology evolves, the addressing of current limitations will further enhance their potential impact [239,241].

## 9. Conclusions

The key pillars of tSCI treatment remain secondary damage reduction (neuroprotection) and rehabilitation. While several promising therapies are being tested in clinical trials, no effective pharmacological treatment currently exists. As a result, neuroprotection continues to rely on optimizing hemodynamic management to ensure adequate spinal cord perfusion pressure and surgical intervention. Emerging technologies show potential to improve real-time spinal cord perfusion monitoring and enable more targeted perfusion strategies.

Although early surgery is widely supported, standardizing surgical protocols, ensuring adequate decompression, and implementing rigorous postoperative evaluations are critical to enhancing clinical outcomes. At present, while stem cell therapies show promise in neuroprotection and repair, their clinical and functional significance remains uncertain, and no general recommendation for their use can be made.

Emerging neuromodulation techniques, such as transcutaneous and epidural stimulation, along with advances in rehabilitation technologies like robotic systems and exoskeletons, are becoming indispensable tools for improving locomotion and overall functionality in individuals with spinal cord injuries. These innovations, when combined with personalized treatment approaches, hold significant potential to transform the management of tSCI.

## Figures and Tables

**Table 1:** Therapeutic strategies in traumatic spinal cord injury.

Reduction in Secondary Damage (Neuroprotection)	Replacement of Lost Cells to Primary and Secondary Damage (Neuroregeneration)	Rehabilitation Strategies (Neuromodulation/Neuroplasticity)
Hemodynamic management Surgical approach Pharmacological neuroprotection	**Stem cells in tSCI** Mesenchymal stem cells Haematopoietic stem cells Neural stem progenitor cells Embryonic stem cells Induced pluripotent stem cells	**Spinal Cord Stimulation** Epidural spinal cord stimulation Transcutaneous spinal cord stimulation **New Technologies in SCI Rehabilitation** Robotic systems and exoskeletons for gait rehabilitation ○ Robot-Assisted Gait Training Systems ○ Overground robotic exoskeletons Virtual reality systems in SCI Rehabilitation Wearable sensors and monitoring technologies

tSCI: Traumatic spinal cord injury. SCI: Spinal cord injury.

**Table 2:** Stem cell types’ comparison.

Stem Cell Type	Potency	Ethical/Availability	Immunogenicity	Primary Therapeutic Mechanism	Paracrine Activity	Tumourgenicity	Clinical Trial Evidence	Safety Profile
MSCs	Multipotent	Minimal concerns; easy to source and scalable	Low (immune-privileged)	Microenvironment modulation	High	Low	Extensive	Low risk
HSCs	Multipotent	Minimal concerns; moderate sourcing; limited scalability	Variable (source-dependent)	Microenvironment modulation	Moderate	Low	Limited	Low risk (autologous use)
NSCs	Multipotent	Minimal concerns; difficult to source, low scalability	Variable (source-dependent)	Cell replacement, microenvironment modulation	Moderate	Low	Moderate	Moderate risk (survival issues)
ESCs	Pluripotent	High concerns; ethically limited, low scalability	High	Cell replacement	Low	High	Minimal	High risk (teratomas, rejection)
iPSCs	Pluripotent	Minimal concerns; technically scalable, requires reprogramming	Low (autologous use)	Cell replacement, microenvironment modulation	Moderate	Moderate to high	Emerging	Moderate risk (genetic issues)

MSCs: Mesenchymal stem cells. HSCs: Hematopoietic stem cells. NSCs: Neural stem cells. ESCs: Embryonic stem cells. iPSCs: Induced pluripotent stem cells.

**Table 3:** Clinical trials on MSCs in chronic SCI.

Study (Year)	SCI Duration	Sample Size for Cell Treatment Group	Severity (AIS)	Phase	Type of Cells	Administration and Dosage	Placebo-Controlled	Control Group	Follow-Up	Notable Outcomes
Bydon et al. (2024) [146]	Subacute and chronic	10	A, B, C	I	Autologous AD-MSCs	Intrathecal; single dose (100 million cells).	No placebo	No control group	96 w (ca. 22 mo.)	No serious AEs; AIS improved in 7/10 participants.
Awidi et al. (2024) [147]	Chronic	20	A, B, C	I/II	Autologous BM-MSCs; allogenic UC-MSCs	Group A: Perilesional BM-MSCs + 3 monthly intrathecal BM-MSCs (100 million cells/dose); Group B: 3 monthly intrathecal UC-MSCs (100 million cells/dose).	No placebo	No control group	22.65 mo. (mean)	No serious AEs; AIS improved in both groups (greater motor recovery in Group A).
Jamali et al. (2023) [148]	Chronic	1	A	Case study	Allogeneic WJ-MSCs	Intrathecal; 6 doses (118 million cells/dose, 1-month intervals).	No placebo	No control group	25 mo.	No serious AEs; AIS improved from grade A to grade C; motor and sensory improvements sustained.
Albu et al. (2021) [149]	Chronic	10	A	I/IIa	Allogenic WJ-MSCs	Intrathecal; single dose (10 million cells).	Yes	Placebo-controlled	12 months (cross-over study with 6 mo. per arm)	No serious AEs; improvement in pinprick sensation.
Vaquero et al. (2018) [150]	Chronic	9 (efficacy), 11 (safety)	A, B, C, D	II	Autologous BM-MSCs	Intrathecal; 3 doses (100 million cells/dose, 3-month intervals).	No placebo	No control group	10 mo.	No serious AEs; AIS improved in 3/10 participants; improvements in sensitivity, motor scores, neuropathic pain, and bladder/bowel function.
Vaquero et al. (2017) [151]	Chronic	10	B, C, D	II	Autologous BM-MSCs	Intrathecal; 4 doses (30 million cells/dose, 3-month intervals).	No placebo	No control group	12 mo.	No serious AEs; improvements in sensory and motor function, better bladder/bowel control, enhanced QoL.
Zhao et al. (2017) [152]	Chronic	8	A	I	Allogenic UC-MSCs + NeuroRegen scaffold	Perilesional; single dose (40 million cells preloaded on scaffold).	No placebo	No control group	12 mo.	No serious AEs; expansion of sensation levels in 5/8 participants; partial motor recovery.
Satti et al. (2016) [153]	Subacute and chronicc	9	A	I	Autologous BM-MSCs	Intrathecal; 2–3 doses (median: 1.2 million cells/kg).	No placebo	No control group	Chronic: median 720 days (range 630–826). Subacute: median 366 days (range 269–367)	No serious AEs.
Oh et al. (2016) [154]	Chronic	16	B	III	Autologous BM-MSCs	Intramedullary (16 million cells) + intrathecal (32 million cells), single dose.	No placebo	No control group	6 mo.	Minor motor improvement in 2/16 participants; DTI revealed new fiber continuity.
Hur et al. (2016) [155]	Chronic	14	A, B, D	I	Autologous AD-MSCs	Intrathecal; 3 doses (30 million cells/dose)	No placebo	No control group	8 mo.	No serious AEs; motor improvement in 5/14 and sensory improvement in 10/14 participants.
Oraee-Yazdani et al. (2016) [156]	Chronic	6	A	I	Autologous BM-MSCs and SC	Intrathecal; single dose (2 million cells/mL, 2 mL total).	No placebo	No control group	Mean 30.6 mo. (range 25–36)	No serious AEs.
Mendonça et al. (2014) [157]	Chronic	14 (12 completed follow-up)	A	I	Autologous BM-MSCs	Intralesional; single dose (5 million cells/cm^3^ lesion volume)	No placebo	No control group	6 mo.	No serious AEs; AIS classification improved in 7/12 participants.
El-Kheir et al. (2014) [158]	Chronic	50	A, B	I/II	Autologous BM-MSCs	Intrathecal; cumulative target dose of 2 million cells/kg, administered monthly (median 4 injections, range: 1–8).	No placebo	Control group received physiotherapy only	18 mo.	No serious AEs; AIS classification improved in 17/50 patients.

## Data Availability

Not applicable.

## Abbreviations

The following abbreviations are used in this manuscript:

tSCI	Traumatic Spinal Cord Injury
SCI	Spinal Cord Injury
SCPP	Spinal Cord Perfusion Pressure
MAP	Mean Arterial Pressure
SCBF	Spinal Cord Blood Flow
SBP	Systolic Blood Pressure
ICU	Intensive Care Unit
AANS	American Association of Neurological Surgeons
CNS	Congress of Neurological Surgeons
GRADE	Grade of Recommendation, Assessment, Development, and Evaluation
AIS	ASIA Impairment Scale
CSF	Cerebrospinal Fluid
ISNCSCI	International Standards for the Neurological Classification of Spinal Cord Injury
ASIA	American Spinal Injury Association
ITP	Intrathecal Pressure
SCPP	Spinal Cord Perfusion Pressure
ISP	Intraspinal Pressure
CSFP	Cerebrospinal Fluid Pressure
CSFD	Cerebrospinal Fluid Drainage
MRI	Magnetic Resonance Imaging
SCIWORA	Spinal Cord injury Without Radiological Abnormalities
DISCUS	Duroplasty for Injured Cervical Spinal Cord with Uncontrolled Swelling
G-CSF	Granulocyte Colony-Stimulating Factor
MP	Methylprednisolone
NASCIS	National Acute Spinal Cord Injury Study
TNF-alpha	Tumor Necrosis Factor alpha
RGMa	Repulsive Guidance Molecule A
ESCs	Embryonic Stem Cells
HSCs	Hematopoietic Stem Cells
MSCs	Mesenchymal Stem Cells
iPSCs	Induced Pluripotent Stem Cells
NSCs	Neural Stem Cells
BM-MSCs	Bone Marrow Mesenchymal Stem Cells
hUC-MSCs	Human Umbilical Cord Mesenchymal Stem Cells
AD-MSCs	Adipose Tissue Mesenchymal Stem Cells
WJ-MSCs	Wharton’s Jelly Mesenchymal Stem Cells
eSCS	Epidural Spinal Cord Stimulation
tSCS	Transcutaneous Spinal Cord Stimulation
SCS	Spinal Cord Stimulation
EMG	Electromyography
IMT	Inspiratory Muscle Training
RAGT	Robot-Assisted Gait Training Systems
ORE	Overground Robotic Exoskeletons
BWST	Body-Weight Support
WISCI	Walking Index Spinal Cord Injury
HAL	Hybrid Assistive Limb
VR	Virtual Reality
HMDs	Head-Mounted Displays
